# Association of inflammatory biomarker abnormalities with mortality in COVID-19: a meta-analysis

**DOI:** 10.1186/s42269-022-00733-z

**Published:** 2022-03-04

**Authors:** Arpita Suri, Naveen Kumar Singh, Vanamail Perumal

**Affiliations:** 1 Department of Biochemistry, SGT Medical College Hospital and Research Institute, Gurugram, Haryana 122505 India; 2 Department of Obstetrics and Gynaecology, All India Institute of Medical Sciences, New Delhi, India

**Keywords:** C-reactive protein, Interleukin-6, D-dimer, Procalcitonin, COVID-19, Survivors, Meta-analysis

## Abstract

**Background:**

COVID-19 outbreak has engulfed different parts of the world, affecting more than 163 million people and causing more than 3 million deaths worldwide due to human transmission. Thus, it has become critical to identify the risk factors and laboratory parameters to identify patients who have high chances of worsening clinical symptoms or poor clinical outcomes. Therefore, the study aims to identify inflammatory markers that can help identify patients at increased risk for progression to critical illness, thus decreasing the risk of any mortality. Our study focussed on the predictive utility of C-reactive protein, Interleukin-6, D-dimer and Procalcitonin in assisting the management of COVID-19 patients with adverse clinical effects. Through literature search in electronic databases, we included the retrospective studies that evaluated the biomarkers among confirmed COVID-19 patients before initiation of treatment and who had a definite outcome (dead or discharged). Biomarkers were expressed in standardized difference in mean value, calculated based on study sizes and mean values between survivors and non-survivors considered the effect size. We carried out a meta-regression analysis to identify the causes of the heterogeneity between the studies.

**Results:**

Number of studies eligible for C-reactive protein, D-dimer and Interleukin-6 markers were eight, seven and four, respectively. Using random effect model revealed that the overall effect size with 95% confidence interval (CI) for C-reactive protein, D-dimer and Interleukin-6 were 1.45 (0.79–2.12) milligrams/litre, 1.12 (0.64–1.59) micrograms/millilitre Fibrinogen Equivalent Units and 1.34 (0.43–2.24) picograms/millilitre respectively was statistically significant (*P* < 0.05) inferring that the mean scores of these marker were significantly higher among the non-survivors compared to the survivors. Two studies were eligible for Procalcitonin marker and there was no heterogeniety (*I*^2^-statistics = 0) between these studies. Therefore, fixed-effect model revealed that the overall effect size (95% CI) for Procalcitonin was 0.75 (0.30–1.21) Nanograms/millilitre was also high among non-survivors.

**Conclusions:**

The study found that serum levels of C-reactive protein, Interleukin-6 and D-dimer showed significant elevation in non-survivors compared to survivors. Raised inflammatory markers aid in the risk stratification of COVID-19 patients and their proper management.

## Background

COVID-19 outbreak originated in Wuhan, Hubei province, China, presenting with pneumonia of unknown aetiology in December 2019. The International Committee on taxonomy of viruses named the Coronavirus study group SARS-COV-2, which belongs to the family Coronaviridae and order Nidovirales (Gorbalenya et al. 2020). It is a zoonotic pathogen that has been transmitted from bats to humans (Li et al. 2020). WHO declared COVID-19 as public health emergency of international concern on 30th January 2020 (Adhikari et al. 2020). The COVID-19 outbreak has engulfed different parts of the world, affecting more than 163 million people and causing more than 3 million deaths worldwide due to human transmission (https://www.who.int/emergencies/diseases/novel-coronavirus-2019/situation-reports.). Thus, the COVID-19 pandemic has become a principal concern to nations worldwide. Thus, it has become critical to identify the risk factors and laboratory parameters to identify patients who have high chances of worsening clinical symptoms or poor clinical outcomes. Studies have suggested that the Cytokine storm has emerged as an essential factor in the etiopathogenesis of fatal effects of COVID-19, predisposing the COVID-19 patients to heightened lung damage called acute respiratory distress leading to higher morbidity and mortality (Bhaskar et al. 2020). The systemic hyperinflammatory syndrome involves the excessive release of pro-inflammatory cytokines advancing multi-organ failure (Fajgenbaum and June 2020) and promoting a prothrombotic milieu (Kaushik et al. 2021). Thus, the study aims to identify inflammatory markers that can help identify patients at increased risk for progression to critical illness, thus decreasing the risk of any mortality. These markers could further help in development of serum based risk stratification algorithm which can assess severity of the disease and help clinicians in recognition of patients at risk of poor clinical outcome.

## Methods

### Inclusion and exclusion criteria

We included the studies if (1) retrospective study analysed the laboratory investigations of rRT-PCR confirmed COVID-19 patients who had a definite outcome (dead or discharged) (2) studies investigating serum C-reactive protein (CRP), D-dimer, Interleukin-6 (IL-6), and Procalcitonin (PCT); (3) blood samples were collected before initiation of treatment. We excluded the studies if (1) language of the abstract or full paper was in any language except English (2) Median, interquartile range of the laboratory investigations in survivor and non-survivors were not present (3) they were case series, case reports, meta-analysis, systematic reviews and editorials.

### Search strategy and selection of articles

Through searching the electronic databases such as Medicine: MEDLINE (through PUBMED interface), EMBASE, Google Scholar, Science Direct and Cochrane library, we identified articles. We included the articles published from December 2019 to May 2020, with search keys “C-reactive protein”, “Interleukin-6”, “D-dime”, “Procalcitonin”, “COVID-19”, and combinations of these keys. We reviewed the full text of the articles to decide their inclusion for meta-analysis.

### Data extraction

We extracted data from the selected studies such as author, publication year, country, study design, outcome, laboratory values. PRISMA flow diagram describes the number of studies screened and included for meta-analysis.

### Statistical analysis

The primary outcome was to assess the levels of various biomarkers such as CRP, D-dimer, IL-6 and PCT. We presented these biomarkers as median and interquartile range (IQR) values in the majority of studies. Therefore, we derived mean values and standard deviations (SD) for the present analysis, prerequisites to calculate the effect size of continuous variables in the meta-analysis. We derived mean and SD values using the formula as recommended in an earlier study (Wan et al. 2014)

$$\begin{aligned} {\text{Mean}} & = \left( {{\text{Median}} + q1 + q3} \right)/{3} \\ {\text{SD}} & = \left( {q3 - q1} \right)/{1}.{35} \\ \end{aligned}$$

Further, we observed all the biomarkers in different units of measurements. Therefore, the mean value of the standardised difference (std. diff) is calculated based on study sizes and mean values between survivors and non-survivors considered the effect size.

We performed a meta-analysis in two stages using Comprehensive Meta-Analysis (CMA) software version 3.0 (evaluation version). We calculated individual study-specific effect size with its 95% confidence interval (CI) in the first stage. We obtained an overall effect size as a weighted (inverse of the effect size variance) average of the individual summary statistics in the second stage. Since each study had different samples, the sampling error variability is likely high in a meta-analysis. The other source of heterogeneity might be due to characteristics of the patients, variations in the treatment, design quality and so on. Therefore, assessing the heterogeneity in meta-analysis is crucial because the presence versus the absence of true heterogeneity (between studies variability) can affect the statistical model. We tested the presence of true heterogeneity using the *Q* test, which follows a chi-square distribution with *k* − 1 degrees of freedom, *k* being the number of studies. When not rejecting the homogeneity hypothesis, we adopted a fixed-effects model. However, the strength of the *Q* statistic depends on the number of studies included in the meta-analysis. Therefore, we used *I*^2^—statistics in percentage values to measure the degree of heterogeneity. While *I*^2^-statistics ≥ 50%, we used a random-effect model.

We depicted the effect size with a 95% confidence interval (CI) for each study and the overall effect size in forest plots. We tested the effect size consistency using sensitivity analysis by leaving one study approach. Using the funnel plot and Egger regression test, we assessed publication bias between the studies. Further, to identify potential factors for heterogeneity, we carried out a meta-regression analysis of the effect size on various covariates such as age, fever rate and cough rate of the patients. For statistical significance, we considered *P* < 0.05.

## Results

**Fig. 1:**
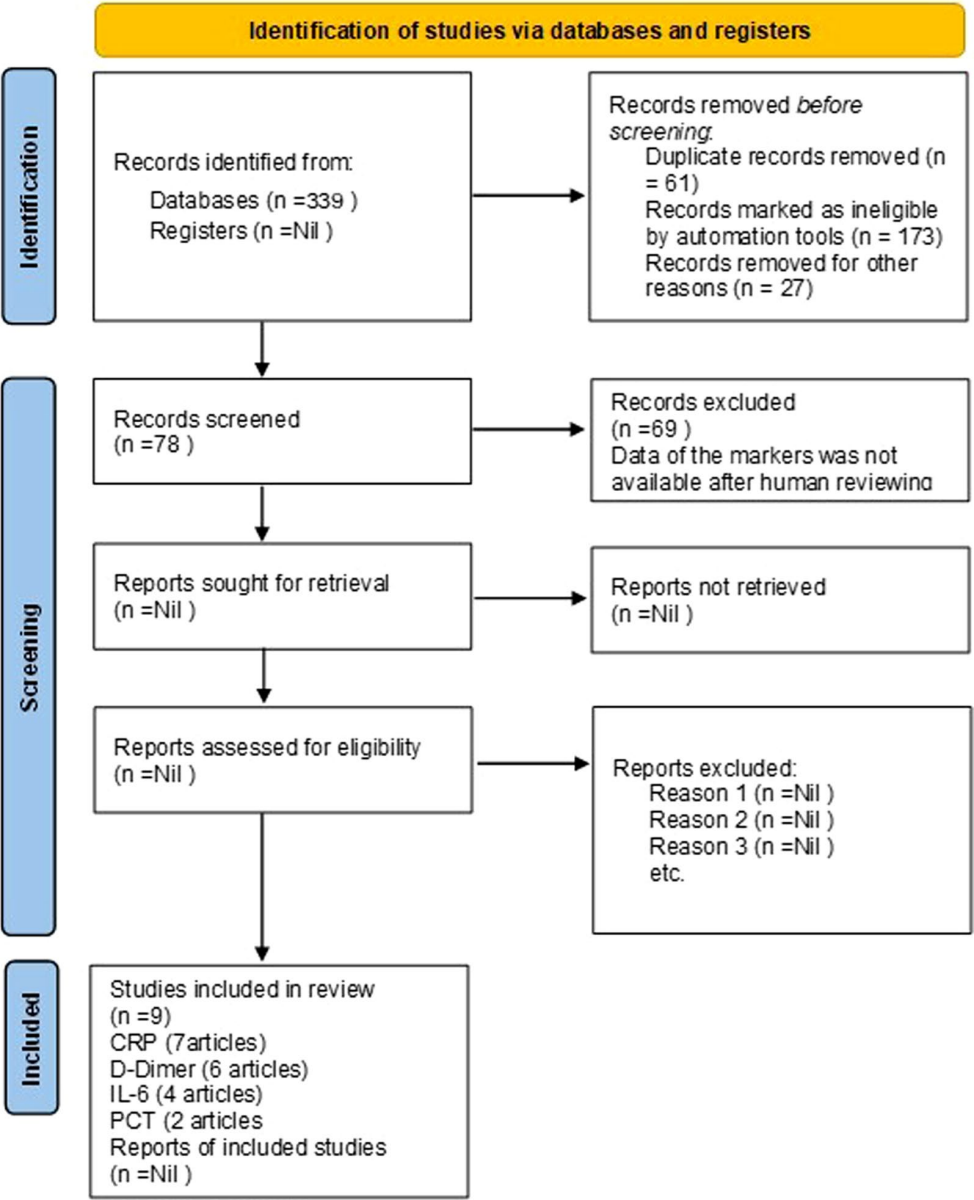
PRISMA flow diagram for systematic review which included searches of databases

PRISMA flow diagram (Fig. 1) shows the stages of the studies screened and included for the analysis. The number of studies selected for markers was seven (Fogarty et al. 2020; Yan et al. 2020; Fan et al. 2020; Deng et al. 2020; Zeng et al. 2020; Chen et al. 2020; Wang et al. 2020), six (Fogarty et al. 2020; Tang et al. 2020; Zhou et al. 2020; Yan et al. 2020; Zhang et al. 2020; Fan et al. 2020), four (Zhou et al. 2020; Yan et al. 2020; Fan et al. 2020; Chen et al. 2020) and two (Yan et al. 2020; Chen et al. 2020) for CRP, D-dimer, IL-6 and PCT markers, respectively. The mean age of these patients varied between 47 and 77 years. The symptoms rates such as incidence of fever (85%), cough (65%), fatigue (46.2%), headache (7%) and diarrhoea (14%) were predominant.

### Effect of CRP markers

**Fig. 2:**
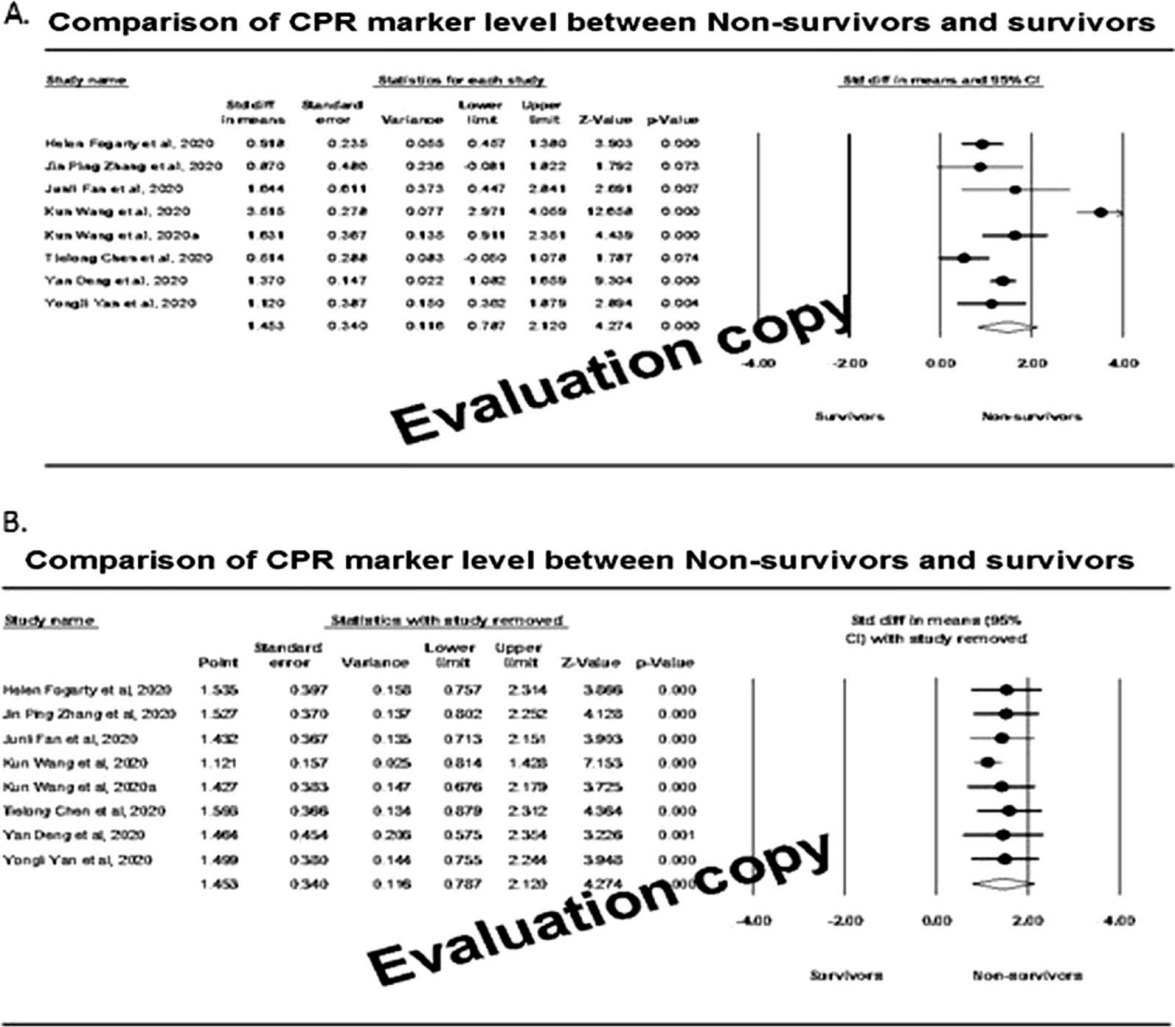
Forest plot (**A**) and sensitivity analysis (**B**) of effect size among survivors and non-survivors

A total of eight studies involving 245 non-survivors and 545 survivors were identified with CRP marker measurement. Individual study-specific analysis indicated that out of eight studies included, six studies (75%) demonstrated that the effect size was statistically significant (*P* < 0.050), inferring that the mean score of CRP marker was significantly higher among the non-survivors compared to the survivors (Fig. 2A). The measures of heterogeneity (*I*^2^) was about 90%, and therefore the random effect model revealed that the overall effect size (95% CI) was 1.45 (95% CI: 0.79–2.12) milligrams/litre. To ensure the consistency of the effect size, we carried out a sensitivity analysis by leaving one study approach. The analysis (Fig. 2B) showed that the effect sizes were between 1.12 and 1.60 and observed within the 95% CI of overall effect size.

**Fig. 3:**
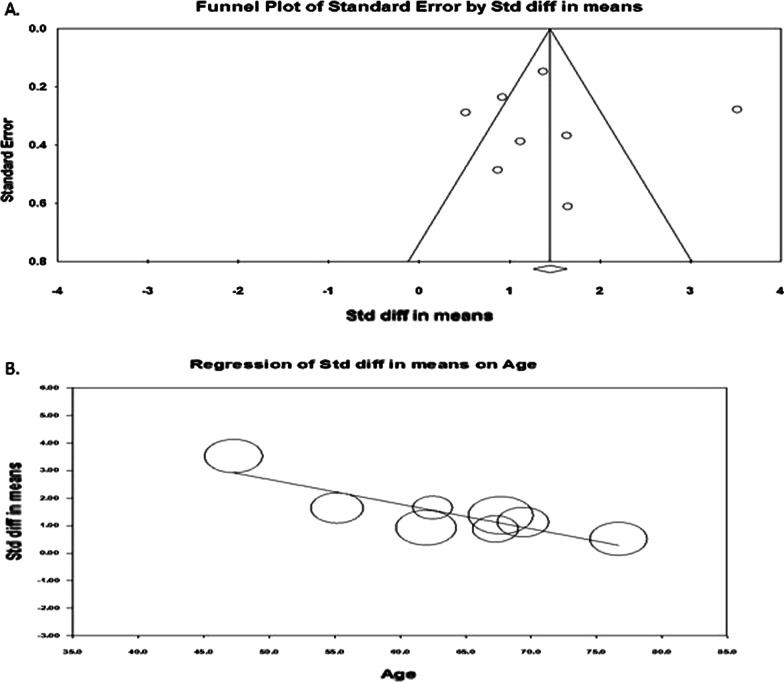
Publcation biasl (**A**) and regressin analysis (**B**) of effect size on age covariate

**Fig. 4:**
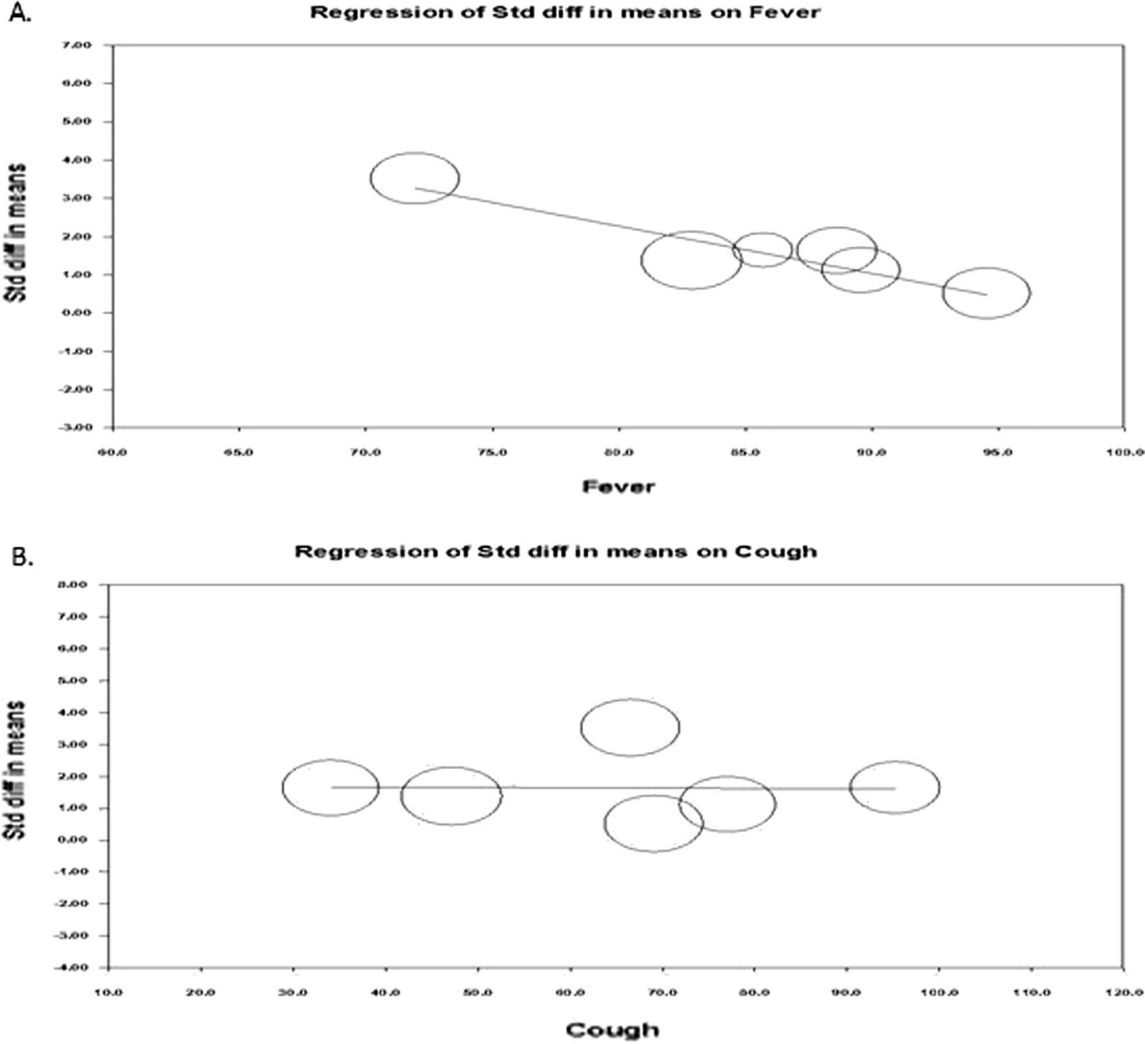
Regressin analysis of effect size on covariates fever rate (**A**) and cough rate (**B**)

We plotted a funnel plot (std.diff versus standard error) to determine publication bias, showing (Fig. 3A) that there was no indication of publication bias. Subsequent egger regression analysis also showed that the intercept was not statistically significant (*P* = 0.944), confirming the absence of publication bias. Since there was high heterogeneity between the studies, we carried out meta-regression to identify possible significant factors among the reported covariates, such as age, fever, and cough rate. Effect size (Fig. 3B) was tend to decrease with increasing age (*R*^2^ = 0.75; *P* < 0.001) and fever rate (Fig. 4A; *R*^2^ = 0.83; *P* < 0.001). The variable cough rate could not establish a significant (*P* = 0.967) factor (Fig. 4B). While carrying out meta-regression with all the three covariates, we observed a similar trend of univariate analysis with *R*^2^ = 0.95; *P* < 0.050.

### Effect of D-dimer markers

**Fig. 5:**
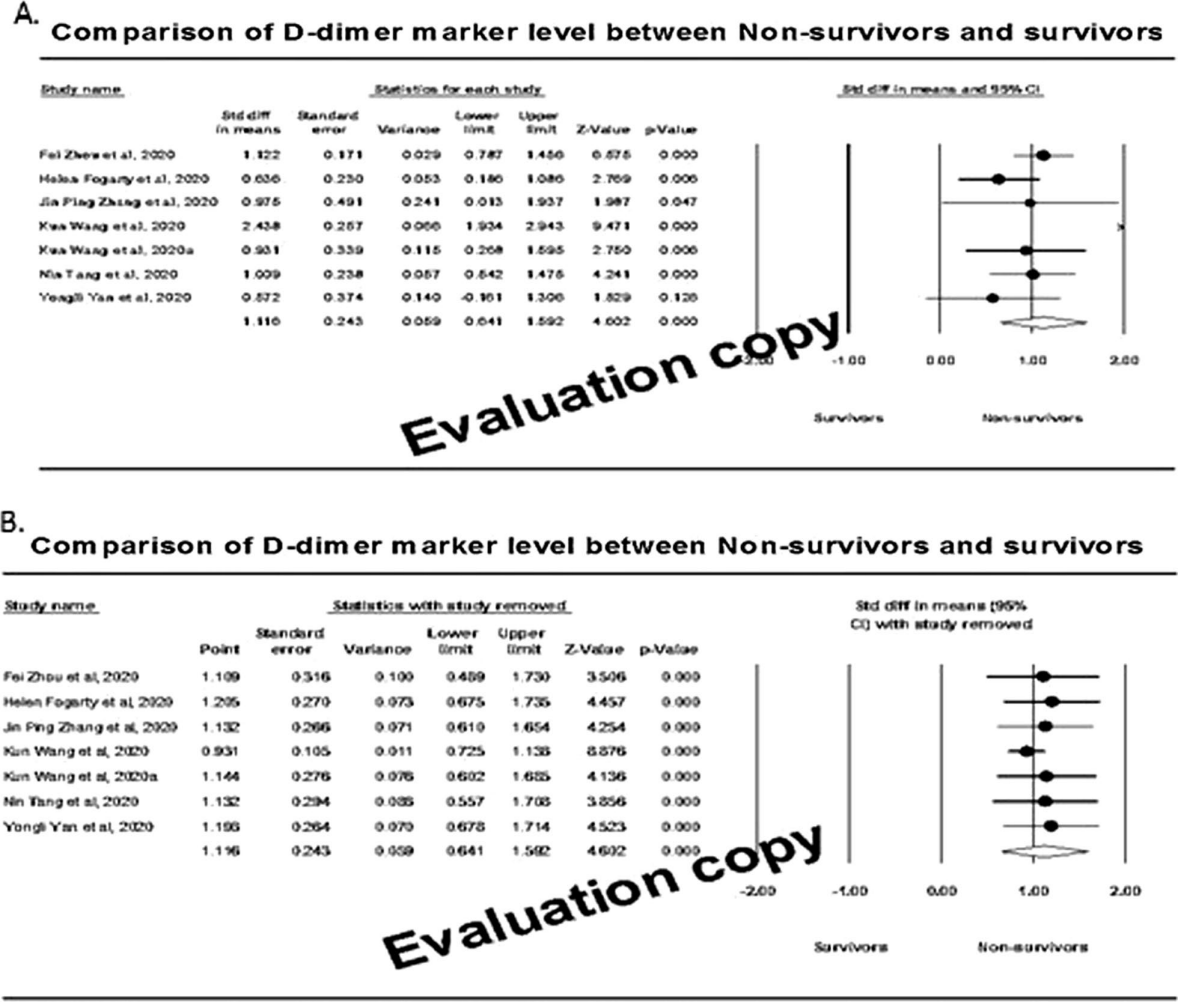
Forest plot (**A**) and sensitivity analysis (**B**) of effect size among survivors and non-survivors

We observed a total of seven studies involving 188 non-survivors and 676 survivors were with D-dimer marker measurement. The effect size of individual studies indicated that out of seven studies included, six (85%) demonstrated that the effect size was statistically significant (*P* < 0.050), inferring that the mean score of the D-dimer marker was significantly higher among the non-survivors compared to the survivors (Fig. 5A). The measures of heterogeneity (*I*^2^) was 82%, and therefore the random effect model revealed that the overall effect size (95% CI) was 1.12 (95% CI: 0.64–1.59) micrograms/millilitre (Fibrinogen Equivalent Units). Sensitivity analysis (Fig. 5B) showed that the effect sizes were between 0.93 and 1.20 and observed within the 95% CI of the overall effect size.

**Fig. 6:**
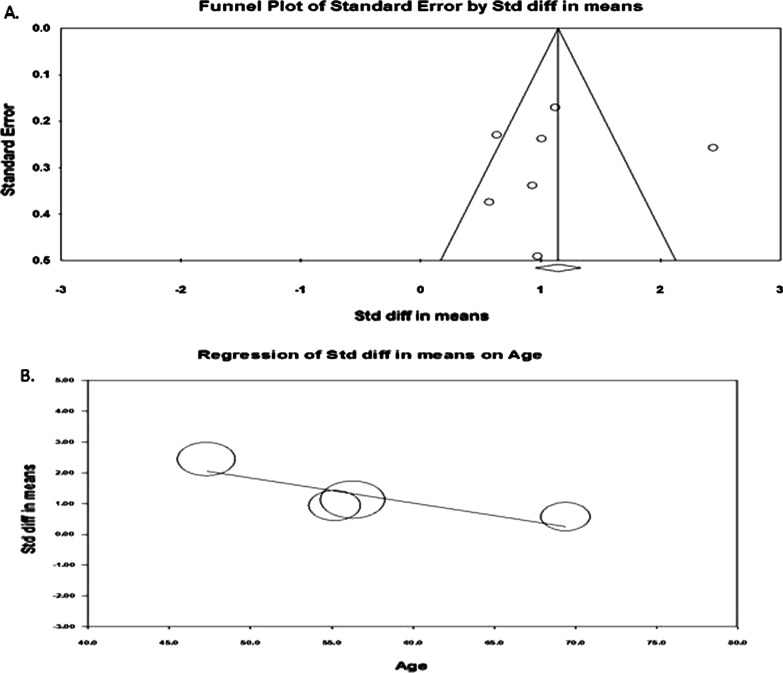
Publcation biasl (**A**) and regressin analysis (**B**) of effect size on age covariate

**Fig. 7:**
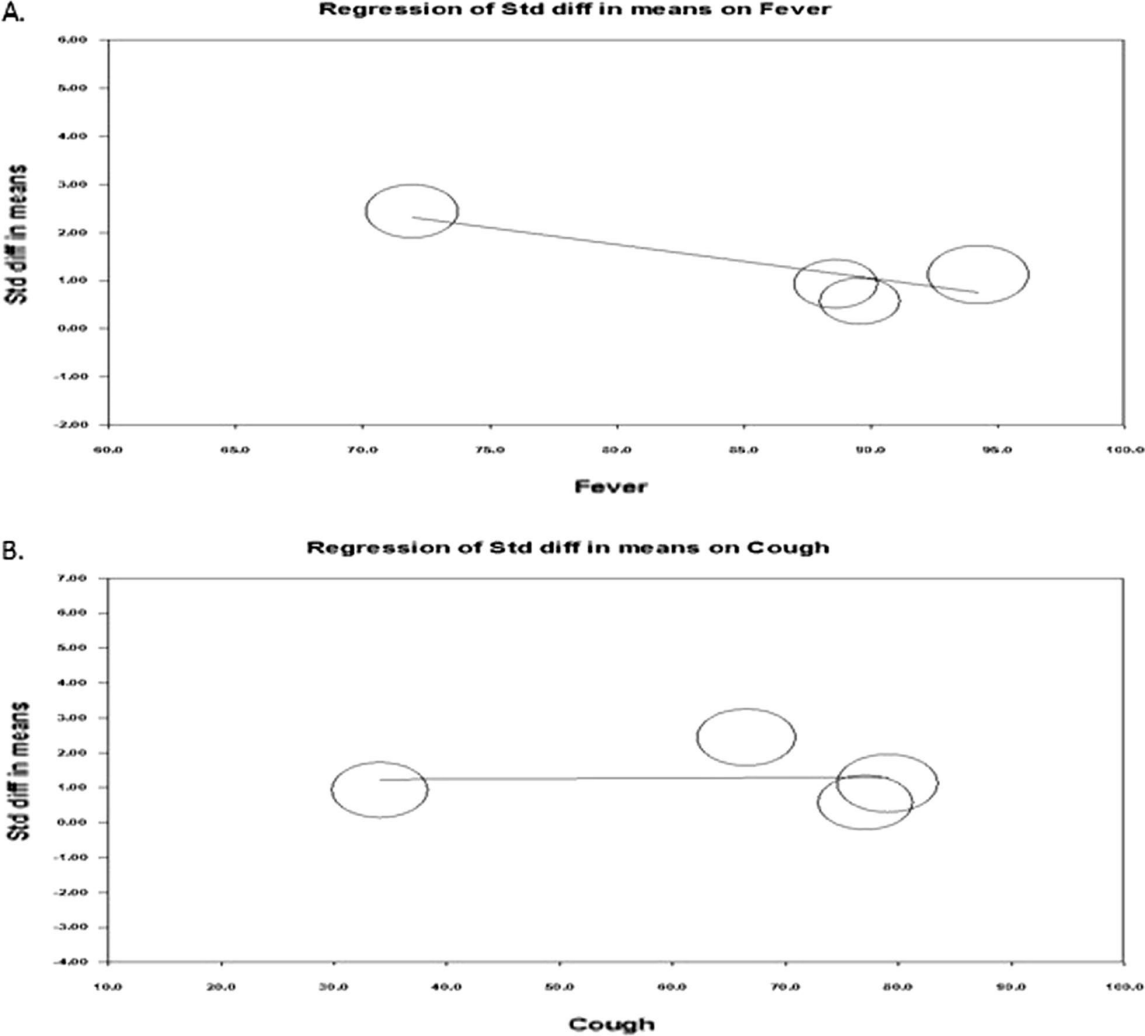
Regressin analysis of effect size on covariates fever rate (**A**) and cough rate (**B**)

The funnel plot showed (Fig. 6A) that there was no indication of publication bias, and subsequent egger regression analysis also showed that the intercept was not statistically significant (*P* = 0.851), confirming the absence of publication bias. Meta-regression of the effect size on covariates showed that effect size (Fig. 6B) was tend to decrease with increasing age (*R*^2^ = 0.76; *P* < 0.008) and fever rate (Fig. 7A; *R*^2^ = 0.70; *P* = 0.012). The variable cough rate did not emerge as a significant (*P* = 0.957) factor (Fig. 7B). Multivariable meta-regression also showed a similar trend of univariate analysis (*R*^2^ = 0.93; *P* < 0.050).

### Effect of IL-6 markers

**Fig. 8:**
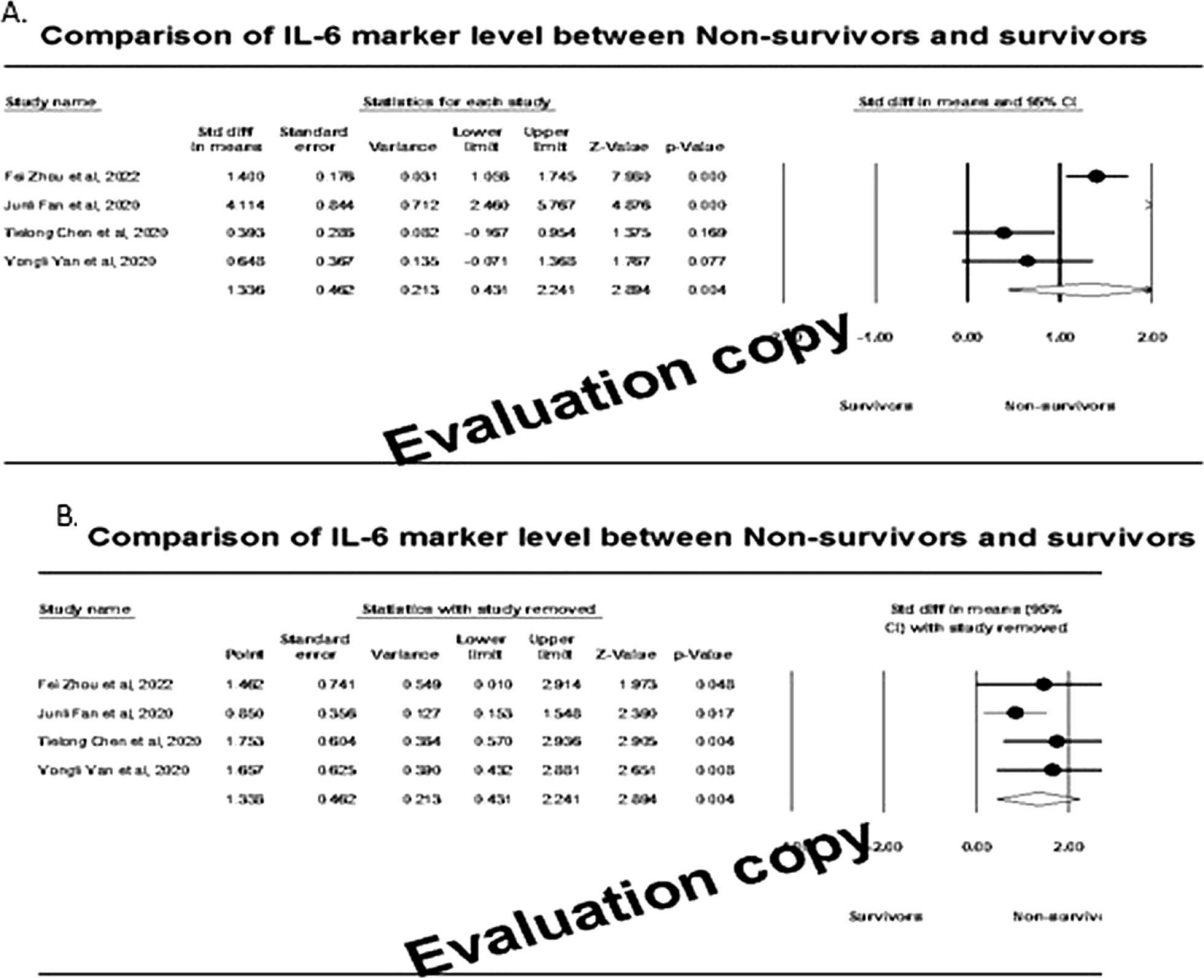
Forest plot (**A**) and sensitivity analysis (**B**) of effect size among survivors and non-survivors

We identified a total of five studies involving 155 non-survivors and 358 survivors with IL-6 marker measurement. Only for four studies, the Il-6 marker level was available. Individual study-specific analysis indicated that out of four studies included, two studies demonstrated that the effect size was statistically significant (*P* < 0.050), inferring that the mean score of the IL-6 marker was significantly higher among the non-survivors compared to the survivors (Fig. 8A). Heterogeneity (*I*^2^) was 87%, and therefore the random effect model revealed that the overall effect size (95% CI) was 1.34 (95% CI: 0.43–2.24) picograms/millilitre. Sensitivity analysis (Fig. 8B) showed that the effect sizes were between 0.85 and 1.75 and observed within the 95% CI of the overall effect size.

**Fig. 9:**
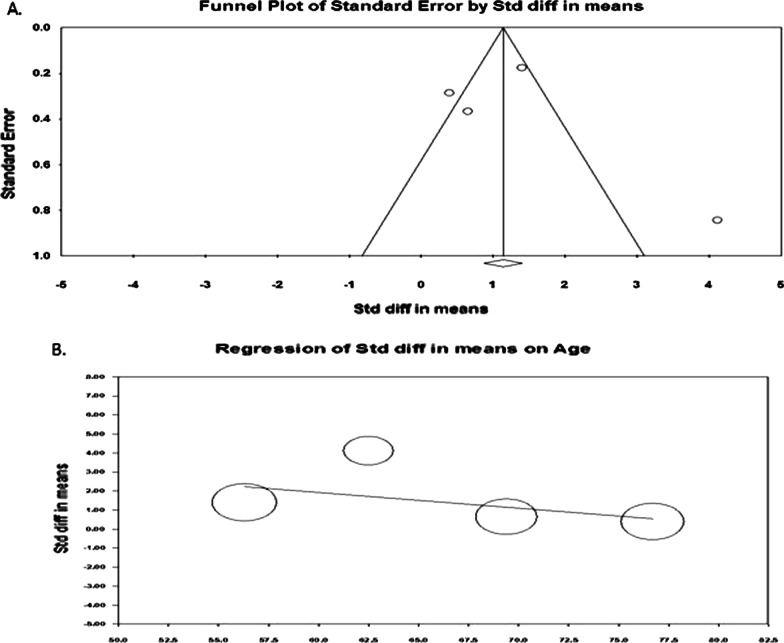
Publcation biasl (**A**) and regressin analysis (**B**) of effect size on age covariate

**Fig. 10:**
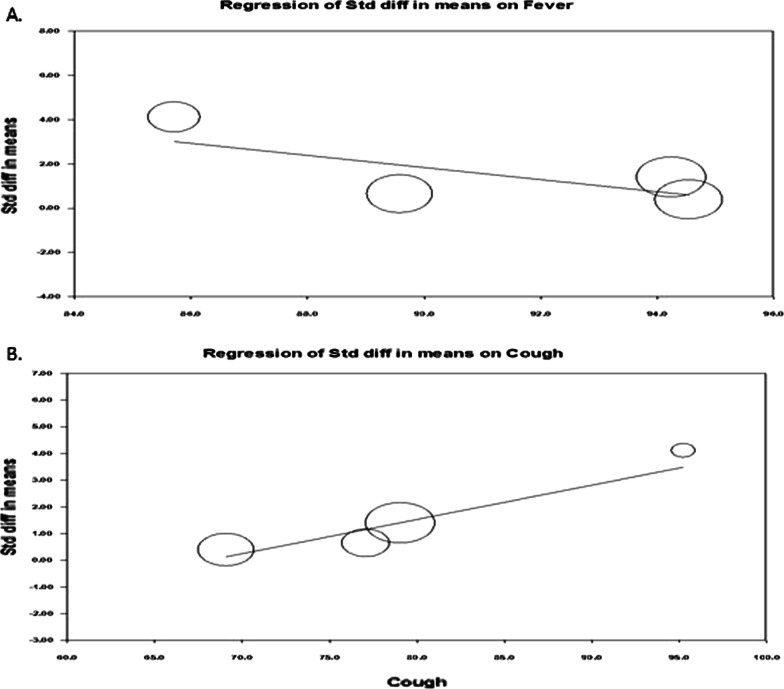
Regressin analysis of effect size on covariates fever rate (**A**) and cough rate (**B**)

The funnel plot showed (Fig. 9A) that there was no indication of publication bias, and subsequent egger regression analysis also showed that the intercept was not statistically significant (*P* = 0.743), confirming the absence of publication bias. In addition, meta-regression of the effect size on covariates showed that the effect size was not significantly influenced by age (Fig. 9B) or fever rate (Fig. 10A). However, the effect size tended to increase with increasing cough rate (*R*^2^ = 0.91; *P* < 0.001), indicating that the IL-6 marker was significantly higher among non-survivors with higher cough rates (Fig. 10B).

### Effect of PCT markers

**Fig. 11:**
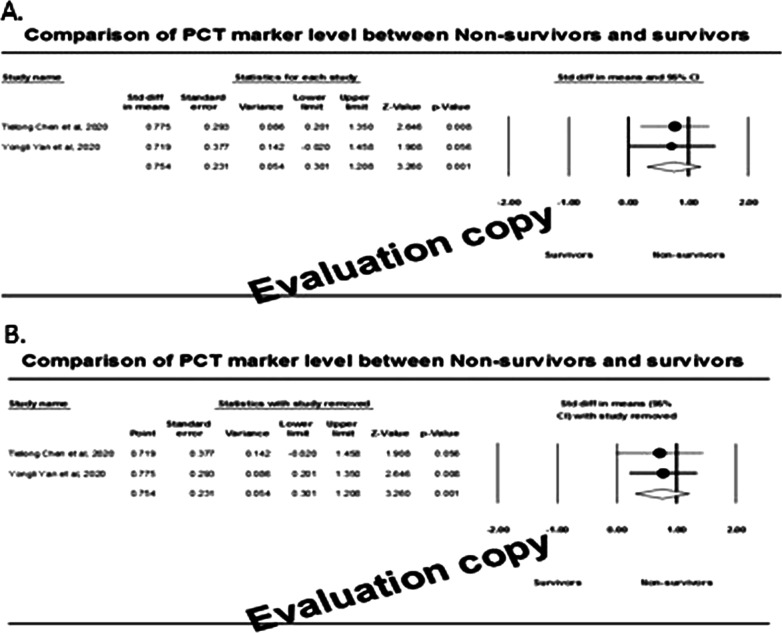
Forest plot (**A**) and sensitivity analysis (**B**) of effect size among survivors and non-survivors

Only two studies involving 58 non-survivors and 45 survivors were found to be with PCT marker measurement. Of these, only one study demonstrated that the effect size was statistically significant (*P* < 0.050), inferring that the mean score of the PCT marker was significantly higher among the non-survivors compared to the survivors (Fig. 11A). Heterogeneity (*I*^2^) was found to be 0%, and therefore the fixed-effect model revealed that the overall effect size (95% CI) was 0.75 (95% CI: 0.30–1.21) nanograms/millilitre. Sensitivity analysis (Fig. 11B) showed that the effect sizes were 0.72 and 0.78 and were observed to be within the 95% CI of the overall effect size.

These two studies were inadequate enough to establish a funnel plot.

## Discussions

### Summary of diagnostic measures

The study found that serum levels of CRP, IL-6, D-dimer and Procalcitonin were significantly elevated in non-survivors compared to survivors. Raised inflammatory markers aid in the risk stratification of COVID-19 patients and their proper management. Our study focused on the predictive utility of these laboratory biomarkers in assisting COVID-19 patients with poor clinical outcome management.

Our knowledge is the first meta-analysis to examine nine studies in the mortality cohort to evaluate statistical analysis, which made our results valid and sound. Exhaustive search strategy and robust statistical analysis promoted the reliability of our study. However, there were a few limitations in our study. First, we included all types of studies, which might influence the effect size due to existence of comorbidity conditions such as hypertension, diabetes, cardiovascular disease, malignancy, pulmonary diseases, chronic kidney, chronic liver disease, and chronic bronchitis. Due to reporting bias of comorbidity conditions, we didn’t add comorbidity conditions as exclusion criteria. Second, due to a limited number of studies included for Serum PCT, we could not assess publication bias. Finally, studies published in foreign languages were not included in our meta-analysis.

### Interpretation

In March 2020, Henry et al. studied laboratory parameters in severity and mortality cohorts of COVID-19 patients. They analysed 21 studies (2984 patients) to assess the association of severity with lab parameters. They concluded markers such as D-dimer, CRP, ferritin, PCT were significantly elevated in patients with severe COVID-19 (Henry et al. 2020). On the other hand, in the mortality cohort, they included three studies and forwarded that these markers were significantly elevated in non-survivors compared to survivors. In May 2020, Aziz M et al. analysed nine studies. They advanced that estimation of interleukin-6 would aid the clinician in prognosticating COVID-19 as it is significantly higher in severe cases than controls (Aziz et al. 2020).

Additionally, they reported that IL-6 levels were associated increased risk of mortality. However, in October 2020, Leisman et al. suggested that role of cytokine release syndrome is questionable in the etiopathogenesis of severe or critical cases of COVID-19 as mean IL-6 in these conditions were significantly lower as compared to that in other inflammatory syndromes such as Sepsis, ARDS and CAR Tcell induced cytokine release syndrome (Leisman et al. 2020). Zheng et al. published a meta-analysis of 16 studies involving 3962 patients (Zeng et al. 2020). They suggested that inflammatory markers such as CRP, IL-6, PCT, ferritin were significantly higher in the severe group than the non-severe group using the random-effects model. Similar to our findings, they postulated that pro-inflammatory cytokine IL-6 was elevated in non-survivors compared to survivors. However, the studies analysed for the meta-analysis were from China, and the data of IL-6 in survivor and non-survivor groups involved only two studies. The importance of elevation of IL-6 can be gauged by the use of Tocilizumab, humanised monoclonal antibody against IL-6 receptor, which has been shown to improve survival and clinical outcome (RECOVERY Collaborative Group 2021). Thus, close monitoring of inflammatory markers can help.

## Conclusions

Our study suggests incorporating these CRP, IL-6 and D-dimer markers to design discriminatory tools and risk stratification tools to adequately identify COVID-19 patients with poor clinical outcomes.

## Data Availability

All the data used in the meta-analyses are available in the listed articles.

## Abbreviations

RT-PCR: Reverse transcriptase polymerase reaction
CRP: C reactive protein
IL-6: Interleukin-6
PCT: Procalcitonin
ARDS: Acute respiratory distress syndrome
CAR: Chimeric antigen receptor
IQR: Interquartile range
SD: Standard deviation
ES: Effect size
std.diff: Standardized difference
PRISMA: Preferred reporting items for systematic review and meta-analyses
CMA: Comprehensive meta-analyses
CI: Confidence interval
